# Fungal Melanin in Plant Pathogens: Complex Biosynthesis Pathways and Diverse Biological Functions

**DOI:** 10.3390/plants14142121

**Published:** 2025-07-09

**Authors:** Hui Jia, Ning Liu, Lu Zhang, Pan Li, Yanan Meng, Wei Yuan, Haixiao Li, Dezeng Tantai, Qing Qu, Zhiyan Cao, Jingao Dong

**Affiliations:** 1 State Key Laboratory of North China Crop Improvement and Regulation, Hebei Agricultural University, Baoding 071000, China; jiahui@hebau.edu.cn; 2 College of Plant Protection, Hebei Agricultural University, Baoding 071000, China; lning121@126.com (N.L.); 20211130056@pgs.hebau.edu.cn (L.Z.); 20232130416@pgs.hebau.edu.cn (W.Y.); 20231280128@pgs.hebau.edu.cn (H.L.); 3 College of Life Sciences, Hebei Agricultural University, Baoding 071000, China; lipan@hebau.edu.cn (P.L.); mengyanan@hebau.edu.cn (Y.M.); 2022614230207@stu.hebau.edu.cn (D.T.); 4 College of Agriculture and Forestry, Hebei North University, Zhangjiakou 075000, China

**Keywords:** fungal melanin, biosynthesis, pathogenicity, stress resistance, plant pathogens

## Abstract

Fungal melanin plays a vital role in the survival, reproduction, infection, and environmental adaptation of plant pathogenic fungi. To develop innovative strategies for managing plant fungal diseases, comprehensive investigations into melanin are imperative. Such research is fundamental to elucidating the mechanistic basis of fungal pathogenesis and holds promise for the design of targeted interventions against melanin-mediated virulence determinants. This review systematically elaborates on the classification of fungal melanin in plant pathogens, provides a detailed analysis of the biosynthetic processes of 3,4-dihydroxyphenylalanine (DOPA) and 1,8-dihydroxynaphthalene melanin (DHN melanins), and reveals the catalytic functions and regulatory mechanisms of key enzymes within these pathways. Melanin modulates fungal virulence by influencing appressorial integrity and turgor pressure formation, thereby participating in the host infection process and the formation of overwintering sclerotia. Melanin provides stress resistance by protecting against extreme environmental factors, including UV radiation and high temperatures. It also has the capacity to absorb heavy metals, which increases pathogen survival under adverse conditions. Furthermore, the review also explores the mechanisms of action of melanin inhibitors that target plant pathogenic fungi, providing a theoretical foundation for developing efficient and environmentally friendly antifungal medications. The complex biosynthesis pathways and diverse biological functions of fungal melanin highlight its significant theoretical and practical importance for elucidating pathogenic mechanisms and formulating scientific control strategies.

## 1. Introduction

**Figure 1:**
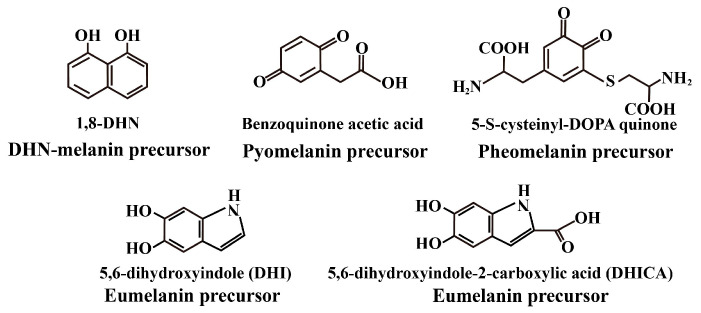
Precursors of each melanin type.

Fungi, melanin is a brown polymer formed by the polymerization of phenolic and indole compounds, widely distributed in animals, plants, and microorganisms. Nicolaus classified melanin into three main types based on nitrogen/sulfur content and color: eumelanin, pheomelanin, and allomelanin [1]. Different chemical precursors lead to variations in melanin type and structure. Eumelanin, a sulfur-free pigment, is polymerized from dihydroxyindole monomers. Pheomelanin contains sulfur and is composed of benzothiazine/thiazole monomers. Allomelanin is nitrogen-free and typically polymerized from phenolic substrates. Based on biosynthetic pathways and intermediate metabolites, Langfelder categorized fungal melanin into four types: 1,8-dihydroxynaphthalene melanin (DHN melanin), pyomelanin, pheomelanin, and eumelanin, with the latter two also referred to as dihydroxyphenylalanine (DOPA melanin) [2]. The structure of each melanin monomer is determined by its precursor molecules, leading to variations in properties among different melanin types (Figure 1). Fossil evidence indicates that melanin is ubiquitous across various organisms, representing an evolutionary product enabling adaptation to environmental stress and co-evolution with host animals predominantly synthesize eumelanin and pheomelanin [3,4,5], while plants produce allomelanin from phenolic monomers [6]. Fungal melanins are the most complex, encompassing all types and often existing as mixed polymeric forms [7]. Fungal melanins are synthesized via laccase-LAC and phenol oxidase-catalyzed polymerization of diverse monomers, resulting in distinct types defined by monomer structure. The most prevalent forms are DOPA-derived and 1,8-dihydroxynaphthalene (DHN)-derived melanins [2]. Despite extensive study, the precise chemical composition of melanin remains undefined, thereby hindering full characterization of its functions and properties.

Melanin, a phenolic polymer ubiquitously expressed in plant pathogenic fungi, orchestrates critical biological processes beyond mere pigmentation. While its role as a ‘fungal armor’ against UV radiation and oxidative stress is well established [2,8], recent evidence highlights its indispensable function in mediating appressorial turgor and host tissue invasion. Beyond its physical protective roles, melanin modulates host immune responses by eliciting antibody production [9]. It also exhibits diverse bioactivities, including antibacterial, antiviral, cytotoxic, anti-inflammatory, and immunomodulatory effects [10,11,12]. These characteristics establish melanin not only as an efficient UV-absorbing antioxidant but also as a promising natural drug carrier, thereby attracting substantial interest in scientific research and practical applications. Studies have established a robust correlation between melanin and the pathogenicity of mechanically invasive pathogens. Melanin-deficient mutant strains lose their pathogenic capacity due to compromised turgor pressure, thereby highlighting the essential role of melanin [13,14]. This review synthesizes current knowledge on melanin biosynthetic pathways in plant pathogenic fungi and their functions in pathogenicity, stress tolerance, and morphogenesis.

## 2. Classification and Biosynthetic Pathways of Fungal Melanin

Fungal melanin is a high-molecular-weight polymer formed via the polymerization of phenolic or indole monomers, often complexed with saccharides or proteins. Melanin participates in diverse fungal physiological processes, including stress tolerance and pathogenicity. As a biopolymer typically insoluble in water and common organic solvents, resistant to acid corrosion, yet sensitive to oxidants, it serves as a UV absorber, antioxidant, and potential natural drug carrier [15]. To date, several distinct types of melanin have been identified in fungi. Based on intermediate metabolites in their synthesis pathways, they are classified into four types: γ-glutaminyl-3,4-dihydroxybenzene (GDBH melanin), catechol melanin, DOPA melanin, and 1,8-dihydroxynaphthalene (DHN melanin) [16]. Among these, DOPA melanin and DHN melanin are the two most important types [2].

**Figure 2:**
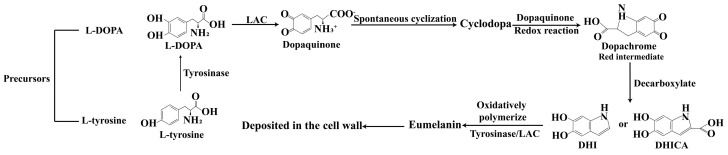
DOPA melanin biosynthesis pathway in fungi. The DOPA melanin biosynthesis pathway begins with two precursors, L-DOPA or tyrosine. L-DOPA is oxidized to dopaquinone by laccase (LAC), while tyrosine is first hydroxylated to L-DOPA by tyrosinase and then oxidized to unstable dopaquinone. Dopaquinone spontaneously cyclizes to form cyclodopa, which undergoes a redox reaction with dopaquinone to produce the red intermediate dopachrome. Dopachrome is decarboxylated to generate 5,6-dihydroxyindole (DHI) or its derivative 5,6-dihydroxyindole-2-carboxylic acid (DHICA). Finally, these indole monomers are oxidatively polymerized by tyrosinase or LAC, forming insoluble eumelanin polymers that deposit in the cell wall.

Fungi such as *Aspergillus niger* and *Basidiomycetes* synthesize DOPA melanin through a biosynthetic pathway analogous to mammalian melanogenesis, utilizing L-DOPA as a substrate [17,18,19]. This pathway can initiate from two potential precursors—L-DOPA or tyrosine. When L-DOPA serves as the substrate, it is oxidized to dopaquinone by LAC. In the case of tyrosine, L-tyrosine is first hydroxylated to L-DOPA by tyrosinase, which is subsequently oxidized to unstable dopaquinone. Dopaquinone undergoes spontaneous cyclization to form cyclodopa, which engages in a redox reaction with dopaquinone to yield the red intermediate dopachrome. Dopachrome is then decarboxylated to generate 5,6-dihydroxyindole (DHI) or its carboxylic acid derivative, 5,6-dihydroxyindole-2-carboxylic acid (DHICA). Finally, these indole monomers are oxidatively polymerized, catalyzed by tyrosinase or LAC, forming insoluble eumelanin polymers deposited in the cell wall (Figure 2). Fungi obtain the initial substrate L-tyrosine from the environment or synthesize it endogenously. Some fungal species can directly utilize exogenous L-DOPA. The human pathogen *Cryptococcus neoformans* represents the most intensively studied model for DOPA melanin biosynthesis [20], as it forms a melanin layer exclusively upon exogenous DOPA supplementation, a process catalyzed by LAC [21]. In contrast, fungi like *Agaricus bisporus* [22,23], *A. niger* and *Sphacelotheca reiliana* [24], and *Dothistroma septosporum* [25] can synthesize natural DOPA melanin without exogenous substrate addition.

**Figure 3:**
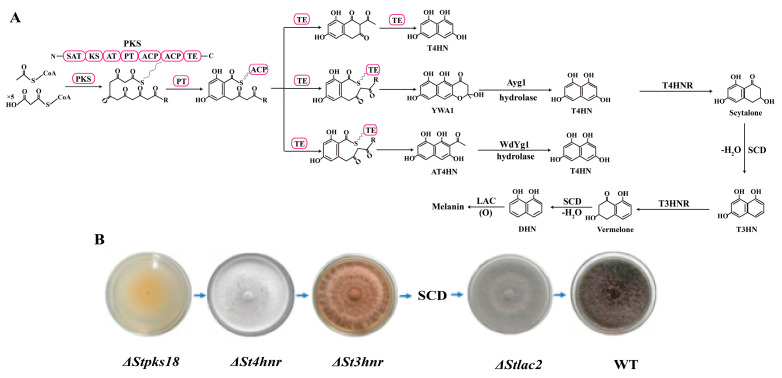
DHN melanin biosynthesis pathway and knockout mutants of partial synthase genes in *S. turcica*. (**A**) The biosynthesis pathway of DHN melanin. The biosynthetic pathway of DHN melanin involves three distinct routes for generating T4HN: (1) the polyketide synthase (PKS) directly releases T4HN as the end product; (2) PKS synthesizes YWA1 as a precursor of T4HN, which is then converted to T4HN through acetoacetic acid elimination catalyzed by Ayg1; (3) PKS produces AT4HN, which undergoes deacetylation to yield T4HN under the catalysis of WdYg1. The pink circles represent the protein domains of PKS. SAT: starter ACP transacylase; KS: ketosynthase; AT: acyltransferase; PT: product template; ACP: acyl carrier protein; TE: thioesterase; T4HN: 1, 3, 6, 8-tetrahydroxynaphthalene; T3HN: 1, 3, 8-trihydroxynaphthalene; T4HNR: tetrahydroxynaphthalene reductase; T3HNR: trihydroxynaphthalene reductase; SCD: scytalone dehydratase; LAC: laccase. (**B**) Identification and functional characterization of genes involved in DHN melanin biosynthesis in *S*. *turcica*. The deletion of key genes in the DHN melanin biosynthesis pathway affects the production of fungal melanin.

DHN melanin is widely distributed in *Ascomycota* and *Deuteromycota*. The canonical DHN melanin biosynthetic pathway was first reported in studies on *Verticillium dahliae* [26]. Investigations into various pathogenic fungi have revealed that endogenously produced precursor molecules—acetyl-CoA or malonyl-CoA—are catalyzed by polyketide synthase (PKS) to form 1,3,6,8-tetrahydroxynaphthalene (T4HN). In fungi, T4HN is synthesized through three distinct pathways. The first pathway involves direct biosynthesis of T4HN by PKS. In the second pathway, PKS produces YWA1, which is converted to T4HN via Ayg1-catalyzed elimination of acetoacetic acid. The third pathway entails PKS-mediated production of AT4HN, followed by WdYg1-catalyzed deacetylation to yield T4HN [27]. T4HN is dehydrated by n sequentially reduced by hydroxynaphthol reductases (1,3,6,8-tetrahydroxynaphthalene reductase, T4HNR; and 1,3,8-trihydroxynaphthalene reductase, T3HNR) and dehydrated by scytalone dehydratase (SCD), forming intermediates scytalone, vermelone, and ultimately DHN. DHN is further polymerized by LAC to form melanin [28]. This entire pathway involves five key catalytic enzymes [29] (Figure 3A). Melanin synthesis is regulated by the transcription factor StMbp1 in *Setosphaeria turcica*, which activates expression by binding the CGCG motif in the *StPKS* promoter, promoting melanin accumulation [30]. The DHN pathway in *B. cinerea* is non-linear; whether melanin forms in sclerotia or conidia depends on the expression of the upstream PKS-encoding genes *bcpks12* (sclerotia) or *bcpks13* (conidia), highlighting the diversity of DHN melanin synthesis across fungi [28]. Furthermore, the key enzymes involved in melanin biosynthesis in plant pathogenic fungi exhibit compartmentalization. Excessive accumulation of the intermediate scytalone is toxic to *B. cinerea*, inhibiting sclerotial and conidial germination [31]. Early-stage enzymes involved in melanin biosynthesis (*BcPKS12/13* and *BcYGH1*) are localized in peroxisomes. Subsequently, scytalone is synthesized within cytoplasmic inclusions and transported via vesicles to the cell surface, where late-stage reactions for DHN melanin formation occur in the cell wall. We previously characterized DHN melanin biosynthesis in *S. turcica*, cloning five biosynthetic genes (*StPKS18*, *St4HNR*, *St3HNR*, *StSCD*, *StLAC*) and one transcription factor (*StMR*). Gene knockout mutants (e.g., *StPKS18*, *St4HNR*, *St3HNR*, *StLAC*) were generated, experimentally confirming their essential roles in melanin biosynthesis [32,33] (Figure 3B).

Fungal melanin is primarily localized within the cell wall, with its polymers exhibiting two major morphologies: spherical particles with diameters of 40–130 nm or irregular sheet-like crystals. These structures are formed by the accumulation of nanoscale subunits and feature highly ordered lamellar arrangements. The natural melanin layer contains pores with diameters of 1–4 nm (rarely up to 30 nm), and the pore size decreases with cell aging. This porous architecture provides channels for trans-wall transport of metabolites (e.g., nutrients and signaling molecules) while blocking the penetration of host defense proteins (e.g., antibodies and enzymes), demonstrating selective permeability. Fungal melanin has a conserved lamellar stacking structure, but the interlayer spacing varies among species: 4.39 Å for *C. neoformans*, 4.15 Å for *Wangiella dermatitidis*, and 4.45 Å for *A. niger* [34].

## 3. The Relationship Between Melanin and Pathogenicity in Plant Pathogenic Fungi

### 3.1. Melanin Participates in Pathogenicity by Influencing Appressorial Integrity and Turgor Pressure Formation

DHN melanin is widely recognized as a virulence factor in numerous pathogenic fungi, playing a crucial role in pathogenesis [35,36,37]. In human pathogenic fungi, melanin demonstrates potent antioxidant activity, scavenging reactive oxygen species (ROS) to subvert host immune surveillance. For example, extracellular melanin secreted by *Fonsecaea pedrosoi* triggers phagocyte activation and enhances their phagocytic efficiency [38], whereas *C. neoformans* melanin evades host phagocytosis by modifying cell surface charge [39]. In *A. fumigatus*, melanin in conidial walls is essential for the proper expression of adhesins and other virulence factors, while inducing only minimal inflammatory cytokine responses to circumvent host antifungal defenses [40,41].

In plant pathogens, DHN melanin serves multiple roles. Beyond protecting fungi from host-derived ROS, it enhances fungal survival and reproduction within the host, exacerbates disease symptoms, and primarily facilitates breaching of host physical defense barriers [42]. Specifically, it improves mechanical penetration at infection sites, such as through the plant cuticle [36]. Many plant pathogens can form melanized infection structures. For pathogens relying on mechanical force for host cell penetration, such as the rice blast fungus *Magnaporthe grisea*, melanin is essential for pathogenicity [14]. During infection, conidia germinate on the plant surface, forming germ tubes that swell at their tips to develop appressoria. In mature appressoria, a dense melanin layer accumulates in the inner cell wall, reducing wall porosity [14]. Septin proteins and actin filaments mediate the maintenance of high internal osmotic pressure and the formation of a penetration peg [43,44,45,46]. Functioning as a selective permeability barrier, the melanin layer facilitates the accumulation of glycerol and intracellular osmolytes within the appressorium, generating extreme turgor pressure. Once turgor pressure reaches a threshold, the histidine-aspartate kinase Sln1 (a pressure sensor) negatively regulates melanin biosynthesis and the cAMP/PKA signaling pathway. This regulation controls melanin synthesis, lipolysis, and glycerol production. Concurrently, it organizes septin proteins and polarity determinants essential for leaf infection. These events trigger the generation of penetration force, leading to rupture of the leaf cuticle and cell wall, and initiating disease [47].

Melanin synthesis is central to the pathogenic process, playing an irreplaceable role in maintaining infection capability. Under normal physiological conditions, pathogens synthesize melanin systematically. Disruption of melanin biosynthesis results in obvious albino phenotypes and loss of pathogenicity [48]. Melanin accumulation is critical for normal appressorial development and function. Mutants with impaired melanin accumulation form defective appressoria that fail to mature properly and cannot trigger the turgor-dependent cell cycle checkpoint. Dysregulation of this checkpoint hinders cytoskeletal reorganization [30]. The cytoskeleton is vital for maintaining cell morphology, intracellular transport, and cell motility; its impaired reorganization severely disrupts normal fungal physiology [49,50]. Concurrently, melanin accumulation defects inhibit penetration peg formation. As the key structure for penetrating host tissue, peg formation failure directly prevents pathogens from breaching host defense barriers. This impedes successful tissue invasion and ultimately impairs pathogenicity [51]. Thus, melanin biosynthesis influences the pathogenic process at multiple critical nodes, serving as a key virulence determinant.

Although melanin is not essential for *B. cinerea* virulence, it plays a role in forming asexual reproductive structures like conidia and sclerotia. Knocking out melanin synthesis genes leads to the accumulation of antimicrobial intermediate metabolites, which may impact fungal development [31]. Conversely, melanin synthesis promotes conidiation in strains with lower melanin content but not in those with high melanin content in *M. grisea* [52]. *Colletotrichum lagenarium* and *M. grisea* synthesize melanin during the appressorial differentiation stage of conidial germination and the late stationary phase of mycelial growth. Two transcription factors, *CMR1* and *PIG1*, are involved in melanin production. In *C. lagenarium*, *CMR1* deletion mutants exhibit defective mycelial melanization. Additionally, *Δcmr1* mutants show impaired expression of the melanin biosynthetic structural genes *SCD1* and *THR1* during melanization, thereby affecting melanin biosynthesis [53]. *Amr1*, a homolog of *Cmr1*, regulates melanin biosynthesis in *A. brassicicola* [54]. Compared to wild-type, *Δamr1* mutants show reduced melanin synthesis and increased sensitivity to UV. In *C*. *gloeosporioides*, deletion of *CgCmr1* or *CgPks1* results in melanin-deficient strains, which exhibit reduced melanin accumulation in appressoria and lower appressorial turgor pressure than wild-type [55,56]. The *ΔCgscd1* mutant shows complete absence of melanin accumulation during appressorial formation or on vegetative hyphae and exhibits reduced hyphal penetration ability [57].

**Figure 4:**
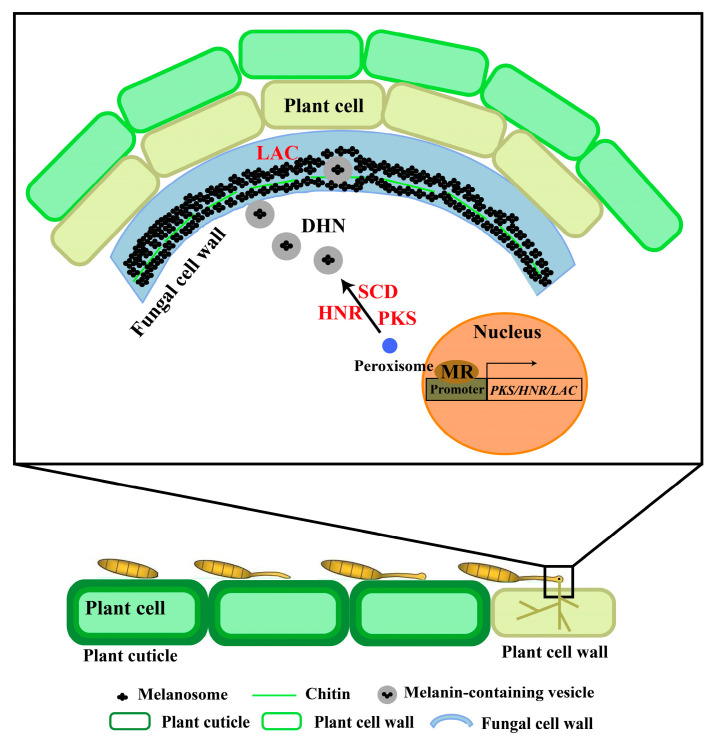
The role of melanin in the pathogenic process of *S. turcica*. During maize infection by *S. turcica*, conidia of the pathogen germinate on the plant surface. Germ tubes form, with their tips swelling to develop into appressoria. In mature appressoria, a dense melanin layer accumulates in the inner cell wall, reducing wall porosity and acting as a selective permeability barrier to promote turgor pressure elevation within the appressoria. Once the turgor pressure reaches a threshold, it regulates melanin biosynthesis. Concomitantly, the appressoria generate infection pegs at their tips, which secrete cell wall-degrading enzymes (e.g., cutinases) to degrade the plant cuticle, enabling mechanical penetration of the underlying epidermal cells. The transcription factor *StMR1* directly binds to the promoter regions of DHN-melanin pathway genes (*StPKS*, *StHNR*, and *StLAC*), thereby modulating melanin biosynthesis levels and influencing appressorium development. PKS: Polyketide synthase; SCD: Scytalone dehydratase; HNR: Hydroxynaphthalene reductase; LAC: Laccase; MR: Transcription factor.

Infection by *S. turcica* commences with spore contact on the maize surface. Following attachment, spores germinate, producing germ tubes that initiate appressorial development [58]. As a key infection structure, appressorial development is regulated by multiple factors, with melanin synthesis playing a central role. Melanin synthesis is intricately linked with DNA replication and autophagy. These processes cooperatively regulate glycerol accumulation and metabolism, enabling the formation of the high turgor pressure structure. Within this regulatory network, the zinc finger protein StMR1 acts as an important transcriptional regulator. StMR1 directly binds to the promoter regions of DHN melanin pathway genes—*StPKS*, *St3HNR*, *St4HNR*, and *StLAC2*—thereby precisely modulating melanin biosynthesis levels and influencing appressorial development [33]. Upon maturation, melanin-mediated modulation of glycerol metabolism establishes high internal osmotic pressure within the appressorium. This high turgor pressure generates the mechanical force necessary for the appressorium to successfully penetrate maize epidermal cells and invade the tissue [59]. Maintaining cell wall integrity is crucial for sustained infection during tissue invasion. *StLAC2*, a member of the LAC-like multicopper oxidase family, plays a dual key role. It participates in DHN-melanin synthesis, ensuring normal appressorial function, and is essential for maintaining cell wall integrity and pathogenicity. This involvement enables effective counteraction of plant defense mechanisms and facilitates successful colonization of maize tissues [32,60] (Figure 4). The infection process of *S. turcica* on maize is a sophisticated biological phenomenon, characterized by the coordinated interplay of multiple physiological processes and the precise regulation of gene expression. From the initial attachment of conidia to the subsequent successful invasion of host tissues and the activation of stress responses, melanin biosynthesis and its associated regulatory factors play indispensable roles at each developmental stage.

### 3.2. Melanin Influences the Formation of Virulence Factors

Beyond regulating virulence by influencing appressorial integrity and turgor pressure, melanin may also affect the formation of other virulence factors [35,36,37,61]. Beyond its role in regulating virulence by maintaining appressorial integrity and turgor pressure, melanin may also affect the biosynthesis of other virulence factors. In *C*. *graminicola*, app yielded 296 mutants with no significant alterationressorial melanization is critical for leaf infection. Notably, melanin is not required for solute accumulation or turgor generation. Instead, it acts as a physical barrier for fungal cell wall-degrading enzymes, protecting cell wall rigidity [62]. The *BRM1* gene in *Alternaria alternata* encodes scytalone dehydratase. It is involved in a two-step biosynthetic pathway. The first step is the dehydration of scytalone to form dihydroxynaphthalene. The second step is the dehydration of vermelone to form trihydroxynaphthalene. Deletion of *BRM1* significantly reduces the production of alternariol toxin (ATX), suggesting a functional correlation between melanin biosynthesis and mycotoxin synthesis. This finding underscores melanin’s critical role as a secondary metabolite in fungal development and pathogenicity [63]. In *Curvularia lunata*, the velvet protein VelB is a core regulator of toxin and pigment metabolism. By interacting with both toxin biosynthesis genes and melanin biosynthetic genes, VelB coordinates these metabolic pathways to enhance pathogen virulence across distinct infection stages and variable environmental conditions [64]. Emerging evidence shows that melanin influences virulence not only in pathogens with melanized infection structures but also plays a positive regulatory role in the pathogenicity of soil-borne pathogens such as *V. dahliae*, which lacks melanized infection structures. The *VdP5CDH* gene has been reported to be involved in melanin biosynthesis, stress tolerance, and virulence regulation in *V. dahliae* [65]. Notably, key melanin biosynthetic genes *Vayg1* and *VdPKS* play critical roles in the infection process of this pathogen [66,67]. The tetraspanin protein VdSho1, functioning as an osmosensor, is critical for plant tissue penetration and melanin biosynthesis in *V. dahliae*. VdSho1 regulates melanin biosynthesis through the Vst50-Vst11-Vst7 kinase signaling module, thereby enhancing the pathogen’s penetration capacity and modulating its pathogenicity [68]. Additionally, during the infection process, melanin in the cell wall of *Aspergillus* species plays a pivotal role in augmenting virulence.

Melanin can chelate Ca^2+^ within phagosomes, thereby inhibiting the Ca^2+^-CaM signaling pathway and blocking host LC3-associated phagocytosis (LAP) [69]. Simultaneously, melanin in the *Aspergillus* cell wall prevents the p22phox subunit of NADPH oxidase from translocating into phagosomes, thereby suppressing NADPH oxidase-dependent LAP activation [70]. Through these mechanisms, melanin effectively attenuates host immune defenses, promoting *Aspergillus* survival and dissemination within the host. This ultimately enhances the pathogen’s pathogenicity and virulence significantly, creating favorable conditions for successful infection.

### 3.3. Melanin Participates in Pathogen Overwintering

In some cases, melanin does not directly participate in the infection process. Instead, it plays a pivotal role in the overwintering survival of pathogens. During the late stages of infection, numerous fungal species generate highly melanized sclerotia, which are compact structures composed of intricately interwoven hyphae. These sclerotia are typically enveloped by an outer melanin layer. This layer confers enhanced stress tolerance through two primary mechanisms: providing a physical barrier against environmental insults and exhibiting antioxidant properties that mitigate oxidative stress. For example, the microsclerotia of *Cordyceps javanica* exhibit 100% survival rates under extreme conditions of high temperature (55 °C) and UV-B radiation [71]. In *B. cinerea*, both sclerotia and conidia display dark pigmentation due to melanin accumulation. The compartmentalization of melanin biosynthetic intermediates serves to prevent autotoxicity, thereby ensuring normal sclerotial development [31]. As dormant structures that enable fungi to withstand harsh environmental conditions, melanized sclerotia exhibit enhanced structural integrity and viability, thereby prolonging their survival in ecological niches such as soil. This adaptive trait ensures that pathogens can re-initiate the infection process when favorable conditions emerge. For plant pathogens in which sclerotia serve as the primary source of inoculum, melanin is therefore critical for maintaining the disease cycle.

### 3.4. Melanin Is Not Involved in Pathogenicity in Specific Fungal Taxa

However, melanin does not participate in the infection process of all plant pathogenic fungi, and in some cases, it may even negatively regulate pathogenicity. Deletion of the melanin biosynthesis genes *CgCmr1* and *CgPks1* in *C*. *gloeosporioides* yielded mutants with no significant alteration in pathogenicity [55]. In *B. cinerea*, deletion of the melanin biosynthesis genes *bcbrn1* and *bcpks13* not only abolished melanization but also enhanced the virulence of mutants on host pepper plants [72]. Additionally, melanin has been shown to be non-essential for virulence in *Sclerotinia sclerotiorum* and *Zymoseptoria tritici*, suggesting that there is no clear correlation between melanin biosynthesis capacity and pathogenicity in these fungal species [73,74].

## 4. The Relationship Between Melanin and Stress Resistance in Plant Pathogenic Fungi

Melanin is not only intricately associated with fungal virulence but also confers enhanced resistance to a range of environmental stresses, including ionizing radiation, UV irradiation, extreme temperatures, and heavy metal toxicity [75].

### 4.1. Resistance to UV Radiation

Melanin-mediated UV resistance stems from a dual mechanism: its conjugated double-bond system efficiently absorbs UV photons, converting them to thermal energy via intramolecular vibration, while its physical architecture scatters incident radiation to reduce cellular penetration [76,77]. This interplay safeguards DNA and enzymatic machinery from photodamage, as evidenced by the 50% lower survival rate of *ΔVdCmr1* mutants compared to wild-type *V. dahliae* under UV exposure [78]. Deletion of *PfMAE* (encoding *ALB1*) in *Pestalotiopsis fici* leads to a drastic reduction in spore tolerance to UV irradiation [79]. Mutants of *P*. *microspora* with a disrupted *pks1* gene exhibit defects in conidial germination and viability [79]. In *B. cinerea*, melanin deposition in conidia and sclerotia confers dark pigmentation, which significantly enhances UV resistance by absorbing and scattering UV radiation, thereby reducing DNA damage [80]. Conidia of *Penicillium digitatum* mutants with knockout of DHN melanin biosynthesis genes (*ΔPdPksP*, *ΔPdAbr1*, *ΔPdArp1*, *ΔPdArp2*, *ΔPdAyg1*) exhibited altered pigmentation. Following UV-C irradiation (522.8 mJ/cm^2^), the viability of these mutants was compromised, and their pathogenicity on Valencia Orange fruits was significantly diminished [81]. UV-induced melanin-deficient mutants of *C. neoformans* exhibited complete loss of pathogenicity [82].

Furthermore, melanin has the property of absorbing a broad spectrum of electromagnetic radiation and converting it into thermal energy. The conjugated double-bond system of fungal melanin plays a pivotal role in this process. It not only efficiently absorbs γ-rays but also transforms radiation energy into chemical energy by altering the electron cloud distribution. This energy conversion mechanism significantly enhances the survival adaptability of fungi in extreme radiation environments [83]. The *A. alternata* isolated from the Chernobyl reactor almost uniformly exhibit extremely high radiation resistance, exemplifying the remarkable survival capabilities of melanized fungi in harsh radiation conditions. Genomic studies reveal that these strains possess highly conserved genomic structures, and their acquisition of radiation resistance may be closely associated with genomic rearrangements, such as chromosomal segment loss and gene amplification. Moreover, experiments simulating the extreme environment of Mars further confirm the potential of melanized fungi to survive under severe extraterrestrial conditions [84]. These findings open up new research directions in the fields of radiation biology and biotechnology. On one hand, the development of radiation protectants and pollutant degradation technologies based on radiation-resistant fungi holds promise for applications in nuclear pollution remediation and occupational protection in high-radiation environments. On the other hand, their application in safeguarding space crops can effectively mitigate the negative impacts of cosmic radiation on plant growth and development. From a theoretical perspective, the energy conversion mechanism of fungi provides a novel paradigm for understanding life survival, offering crucial theoretical insights into the exploration of life forms in the universe and strategies for adaptation to extraterrestrial environments.

### 4.2. Heat Resistance

Melanin plays a vital role in fungal survival under extreme temperatures by acting as a thermal buffer through heat absorption and scattering. Upon exposure to high temperatures, melanin absorbs thermal energy, thereby reducing direct heat transfer to the cell interior and mitigating increases in intracellular temperature. This mechanism prevents denaturation and inactivation of cellular macromolecules (e.g., proteins, nucleic acids) and maintains normal physiological functions. Additionally, high temperatures can induce excessive production of reactive oxygen species (ROS), such as superoxide anions and hydrogen peroxide, within fungal cells. This ROS burst causes oxidative damage to cellular components, including lipids, proteins, and DNA. Melanin exhibits antioxidant activity, scavenging ROS or inhibiting their production to protect cells from oxidative stress damage [42]. As an integral component of the fungal cell wall, melanin interacts with other wall constituents to enhance structural stability. Under high-temperature conditions, the cell wall is susceptible to damage, and melanin helps maintain its integrity, thereby preventing cell death due to wall rupture [40]. Additionally, melanin acts as a physical barrier, restricting the entry of harmful exogenous substances and maintaining a relatively stable intracellular environment [85].

### 4.3. Heavy Metal Adsorption

Melanin exhibits a unique chemical architecture and properties that enable it to adsorb and immobilize heavy metal ions. The melanin molecule contains diverse functional groups (e.g., hydroxyl, carboxyl, and amino groups), which can undergo complexation and chelation reactions with heavy metal ions, thereby binding them to the melanin structure [42]. Studies have demonstrated that microbial melanin, owing to its rich functional groups, can be employed for heavy metal adsorption in aqueous systems. Melanin-based nanomaterials exhibit notable adsorption capacities for Cd^2+^, Cu^2+^, and Pb^2+^, with the adsorption capacity for Pb^2+^ reaching up to 45 mg/g [86]. Plant pathogenic fungal melanin is proposed to adsorb and immobilize heavy metals through analogous mechanisms, thereby reducing the activity and bioavailability of heavy metal ions and mitigating their toxicity to fungal cells. Melanized pseudosclerotia of *Phellinus weirii* exhibit adsorption of soil heavy metal ions, including Al^3+^, Cu^2+^, Fe^3+^, Mn^2+^, Ni^2+^, Pb^2+^, and Zn^2+^ [87]. The binding of fungal melanin to soil heavy metals not only reduces or eliminates their toxicity in contaminated soils but also facilitates the reconstruction of microbial diversity, thereby enabling bioremediation. Soil remediation based on the principle of melanin chelating and inactivating heavy metal ions represents an environmentally friendly technology that has garnered growing attention [87].

Heavy metals pose significant stress to plant pathogenic fungi. Upon detection of heavy metal stress, fungi activate diverse physiological and molecular response mechanisms, among which melanin biosynthesis plays a crucial role. Melanin synthesis enables fungi to mitigate heavy metal stress, thereby enhancing their survival in adverse environments. Melanin functions as a physical barrier, restricting the entry of heavy metal ions into fungal cells [88]. Furthermore, its antioxidant properties enable the scavenging of excessive ROS generated intracellularly under heavy metal stress, thereby protecting cells from oxidative damage [89]. Fungal melanin can also safeguard pathogens against oxidative stress by scavenging free radicals and mitigating oxidative bursts in host cells.

## 5. Mechanisms of Action of Melanin Inhibitors Against Plant Pathogenic Fungi

Plant pathogenic fungal diseases pose significant threats to global agricultural production, causing substantial losses in crop yield and quality. Traditional chemical fungicides have been pivotal in managing these diseases, yet their prolonged use has triggered critical issues, including the emergence of pathogen resistance, environmental contamination, and risks to human health. Thus, the development of novel, efficient, and eco-friendly antifungal agents is of paramount importance. DHN melanin, a critical constituent of pathogenic fungal cells, is pivotal for fungal pathogenicity. Therefore, the design of fungicides targeting the inhibition of fungal melanin biosynthesis presents substantial applied value.

Within the DHN melanin biosynthetic pathway, multiple key enzymes are involved, and inhibitors can target these enzymes to disrupt synthesis. PKS is essential for polyketide chain synthesis in the DHN pathway. Melanin inhibitors can bind to the active site of PKS, altering its conformation and inhibiting catalytic activity, thereby blocking polyketide chain synthesis. This prevents the formation of melanin precursors, ultimately halting DHN melanin production [90,91]. Tricyclazole is a classic and widely used melanin synthesis inhibitor that specifically targets the activity of 3HNR. In the DHN melanin biosynthetic pathway, T3HN is reduced by 3HNR to form DHN, which subsequently undergoes oxidative polymerization to yield melanin [92]. Without normal melanin synthesis, the appressorium fails to form a functional melanin layer in its cell wall. Consequently, the fungus cannot penetrate the plant epidermis, and the infection process is suppressed. Currently, tricyclazole, fenoxanil, benzothiostrobin (probencarb), and carpropamid are widely employed as antifungal agents for controlling rice blast [14,93]. Application of these compounds during the early stages of disease development has been shown to effectively reduce disease incidence and preserve rice yield and quality.

Even if melanin precursors are synthesized normally, inhibitors can target enzymes involved in the oxidative polymerization step, such as LAC or tyrosinase, to prevent the final formation of melanin [94,95]. For example, certain inhibitors bind to the copper ion active center of LAC, decreasing its oxidizing capacity. This prevents the oxidative polymerization of DHN into melanin, thereby attenuating pathogen virulence.

## 6. Conclusions

This review systematically summarizes the multifaceted roles of fungal melanin in plant pathogens, including its biosynthesis, pathogenicity, stress resistance, and inhibition strategies. Fungal melanin was categorized into four types based on its biosynthetic pathways and intermediate metabolites. Among these, DOPA and DHN melanin are the two most important types. DOPA and DHN melanins utilize distinct pathways, with key enzymes like PKS and LAC governing their synthesis. Melanin ensures virulence by maintaining appressorial turgor and mediates stress resistance through UV absorption, thermal buffering, and heavy metal chelation. Inhibitors targeting melanin biosynthesis, such as tricyclazole, provide an eco-friendly approach to antifungal strategies. Future research should focus on clarifying the regulatory networks of melanin biosynthesis and exploring its functional plasticity to develop more precise antifungal strategies and address agricultural and environmental challenges.
